# Dental students’ attitudes toward community service: a pre–post survey study of a service-learning experience in Chile

**DOI:** 10.1186/s12909-026-10109-w

**Published:** 2026-08-12

**Authors:** Carolina Leiva Hernández, Felipe Rodrigo Aguilera, Verónica Aravena-Castillo, Viviana Hidalgo V., Javier Farías Orellana, Adriana Stuardo-Parada

**Affiliations:** 1 Faculty of Dentistry, Universidad San Sebastián, Valdivia, Chile; 2 School of Dentistry, Faculty of Medicine, Universidad Austral de Chile, Valdivia, Chile

**Keywords:** Attitude of Health Personnel, Community-Based Education, Dental Education, Dental Students, Service-Learning

## Abstract

**Background:**

Dental Education has traditionally emphasized the technical and biological aspects of oral healthcare, often overlooking the social determinants of health. Service-Learning has emerged as a pedagogical strategy that blends community engagement with academic goals and has been associated with improved attitudes toward community service among health-professions students; however, evidence from Latin America, and from Dental Education in Chile in particular, remains scarce. This study assessed dental students’ attitudes toward community service before and after participation in Service-Learning activities.

**Methods:**

A pre–post survey study was conducted between August and December 2025 among undergraduate dental students at a Chilean University who took part in community-based educational activities. Community service attitudes were measured using a Spanish adaptation of the Community Service Attitudes Scale (CSAS), a 10-subscale Likert-type instrument administered online three weeks before and three weeks after the intervention. Internal consistency was good (Cronbach’s α = 0.885 pre-test; 0.957 post-test). Of 162 students who completed the pre-test, 77 were matched to post-test responses and included in the analysis. Changes across the 10 subscales were analyzed using repeated-measures ANOVA, and effect sizes were calculated as Hedges’ g.

**Results:**

Most participants were female (64%) and had no prior community service experience (69%). Significant changes were observed in three subscales: norms increased from pre- to post-intervention (LS Mean 4.44 vs. 4.56; *p* = 0.035), empathy showed the largest change (3.18 vs. 4.34; *p* < 0.001), and perceived costs decreased (4.21 vs. 3.44; *p* < 0.001). The remaining seven subscales showed no significant change. Subgroup analyses showed that the reduction in perceived costs was significantly smaller among junior students than among intermediate and senior students (*p* < 0.05).

**Conclusions:**

Participation in Service-Learning was associated with improvements in empathy and social norms and with a reduction in perceived barriers to community engagement among dental students. These findings support the early incorporation of community-based experiences into the Dental Curriculum. This study adds evidence from a Latin American setting where such data are scarce, highlighting the global relevance of Service-Learning for developing socially responsible dental professionals.

**Supplementary Information:**

The online version contains supplementary material available at https://doi.org/10.1186/s12909-026-10109-w.

## Background

Contemporary Dental Education aims to prepare graduates with a comprehensive set of competencies, including clinical expertise, critical thinking, communication, leadership, and the ability to deliver person-centered care [1]. Achieving these outcomes requires not only the acquisition of technical skills but also exposure to real-world clinical environments that foster professional judgment and social awareness before entering professional practice [2, 3].

However, dental training has traditionally emphasized the biological and technical aspects of oral healthcare, often overlooking the social, economic, and cultural determinants that influence health outcomes [4, 5]. This approach may limit the graduates’ ability to respond effectively to diverse community needs and contributes to persistent inequalities in the access to care and quality of oral health services [6]. In this regard, developing socially responsible dentists has been identified as a fundamental goal of Dental Education, as dentists are expected not only to treat individual patients but also to address broader community oral health needs, particularly among underserved and vulnerable populations [7].

To address these limitations, Community-Based Dental Education (CBDE), also referred to as outreach or Service-Learning, has been increasingly incorporated into dental curricula worldwide [2, 8]. Among these approaches, Service-Learning is a structured pedagogical strategy that combines community service with academic objectives through reciprocal partnerships and critical reflection [9–11]. It is characterized by a balance between service and learning, active collaboration with community partners, curricular integration, and structured reflection —the latter being essential for connecting service experiences with academic content and deepening students’ understanding of the social context of health [12, 13].

Evidence in education of health professions, particularly in Dentistry, indicates that Service-Learning can improve students’ attitudes toward community service and enhance key competencies such as empathy, teamwork, communication, and critical thinking [10, 13, 14]. It also contributes to a greater understanding of the dentist’s role in primary care, strengthens ethical awareness, and promotes a patient-centered approach to care [2, 6]. Notably, these gains extended even to students with no prior interest in community service, underscoring the formative potential of the methodology [15–17]. This evidence, however, has methodological limitations: study designs and instruments vary widely, and the frequent lack of control groups limits comparability across findings [13, 14].

Furthermore, research on Latin American Dental Education contexts is limited, raising the question of whether these findings can be applied to settings with different social, cultural, and institutional characteristics [18, 19].

Such international evidence is particularly relevant in middle-income countries, where structural reforms in higher education are creating new institutional conditions for formally integrating Service-Learning into health professions curricula. In Chile, research on this topic has primarily concentrated on other health disciplines, with little emphasis on Dentistry [12, 18, 19]. The Chilean higher education system provides an appropriate institutional setting for this study. Since 2018, *Vinculación con el Medio* (community engagement) has been made a mandatory component of institutional certification for all Chilean Universities, with full implementation set for 2025 [20, 21]. This policy framework provides an opportunity to formally include Service-Learning into health professions courses. Furthermore, Chile has persistent socioeconomic disparities in access to dental care [22], emphasizing the importance of training professionals with a strong sense of social responsibility. However, systematic studies assessing the educational impact of Service-Learning on Dental Students’ attitudes are scarce [14].

This paucity of context-specific evidence constrains the formal incorporation of Service-Learning into the Dental Curriculum and limits understanding of its contribution to the comprehensive preparation of future dentists. Accordingly, the aim of this study was to assess changes in Dental Students’ attitudes toward community service, measured with a structured survey administered before and after participation in Service-Learning activities at a University in Southern Chile. Based on prior evidence from health professions education, it was hypothesized that participation would be associated with more favourable attitudes toward community service.

## Methods

A quasi-experimental pre-post study was conducted to assess changes in Dental Students’ attitudes toward community service. The study was carried out between August and December 2025 as part of community-based educational activities offered by a dental program in Chile. This study was reported in accordance with the TREND guidelines for nonrandomized evaluations.

Ethical approval was obtained from the Scientific Ethics Committee of Universidad San Sebastián, Chile (Approval No. 160 − 25). All participants provided informed consent prior to enrollment.

Participants were undergraduate Dental Students who took part in the community-based educational activities during the study period. Following a convenience sampling strategy, the entire cohort registered across the three academic levels in which these activities were integrated was included (*N* = 211); because attendance was not mandatory, students could join or leave at any time. No a priori power analysis was performed to determine the sample size, as the number of participants was fixed by enrolment in these academic levels. Instead, the adequacy of the available sample was assessed in terms of precision for estimating a population proportion: a minimum of 137 respondents was required to estimate a proportion with a 95% confidence level, a ± 5% margin of error, and the most conservative expected proportion of 0.5. Students were informed about the study aims and procedures, and their participation was entirely voluntary. Each student was invited to complete an online survey at two time points: before the activities began (pre-test) and after they concluded (post-test). To be included, a student had to be enrolled at one of these levels and complete both instruments, with pre- and post-test responses that could be matched. Matching relied on self-generated identification codes, which each participant produced from fixed personal information known only to them, preserving anonymity while allowing the two responses to be paired. Non-response was defined as failure to complete the survey, and incomplete responses were discarded. Students whose responses could not be paired across the two time points were excluded from the analysis.

The intervention consisted of educational activities carried out through the institution’s community outreach program. It comprised two complementary components: an outreach component, conducted at organizations external to the university, and a clinical component, conducted at the university’s facilities. The outreach component took place at preschools and a center for elderly care, both located near the university, which allowed the institutional community engagement program to coordinate student transportation to both sites. At these settings, students delivered oral health promotion and preventive education tailored to each target population: at the preschools, education on diet, toothbrushing technique, and the management of deleterious oral habits was directed at the children’s parents; at the elderly care center, activities focused on denture care and maintenance, along with oral health promotion adapted to older adults. The clinical component was conducted at the university, where each population received care appropriate to its needs: for the preschool-aged children, it comprised clinical examination, detection of deleterious oral habits and caries lesions, and basic restorative treatment; for the older adults, it comprised total and partial denture maintenance, dental prophylaxis, and dental scaling. Activities took place over 15 weeks, with two weekly sessions of approximately three hours each, allocating an equivalent amount of time to the outreach and clinical components. Through this combination of community-based and clinical activities, the program aimed to foster students’ social responsibility, empathy, and understanding of community health needs.

Data were collected using a web-based questionnaire adapted from the Community Service Attitudes Scale (CSAS) developed by Shiarella et al. [23]. The instrument was translated into Spanish and reviewed by a bilingual native speaker to ensure semantic equivalence, and it was administered to participants in Spanish. Additional file 1 presents the original English items together with the Spanish version used in this study. A pilot study was conducted with 24 speech therapy students — a health sciences cohort not involved in the main study, to avoid sensitizing the target sample — to assess the reliability of the Spanish version, which showed excellent internal consistency (Cronbach’s α = 0.929). Participants’ feedback on item clarity and comprehension was collected during piloting; given the excellent internal consistency, no items were modified. Subscale-level reliability for the pilot is reported in Additional file 2.

The CSAS is a Likert-type scale comprising ten subscales: awareness, actions, ability, connectedness, norms, empathy, costs, benefits, seriousness, and intention. In the present study, all ten subscales originally proposed for the CSAS were retained, comprising 46 items and including empathy as a distinct subscale, rather than the empirical eight-factor solution reported by Shiarella et al. [23]. Consistent with Schwartz’s model, the intention dimension, which corresponds to the behavioural response stage of the model, was measured and analysed pre-post in the same manner as the attitudinal subscales. Responses were recorded on a five-point Likert scale ranging from 1 (strongly disagree) to 5 (strongly agree). Sociodemographic information was also collected, including age, sex, place of residence (urban/rural), prior participation in community activities, and academic level.

The final Spanish version of the CSAS was administered online at two time points: before (pre-test) and after (post-test) participation in the community-based activities. The pre-test was administered three weeks before the start of the activities and the post-test three weeks after their completion. Internal consistency was satisfactory for all ten subscales at both time points, with Cronbach’s α ranging from 0.71 to 0.91 at pre-test and from 0.83 to 0.93 at post-test (Additional file 3), supporting the reliability of the ten subscales in this sample. Internal consistency for the overall scale was 0.885 at pre-test and 0.957 at post-test.

To reduce potential sources of bias, participation was optional and responses were collected anonymously. To reduce social desirability bias, students were explicitly told that their responses would have no effect on their academic standing or grades.

Data were analyzed using R (version 4.3.3). Descriptive statistics were calculated for each variable. Item responses were coded numerically, and composite scores for each of the ten CSAS subscales were obtained by averaging the corresponding item responses; these scores were treated as continuous variables. Differences between pre- and post-test scores across the ten subscales were examined using repeated-measures ANOVA, with participant included as the error stratum to account for the within-subject design. Least-squares (estimated marginal) means and 95% confidence intervals were obtained with the emmeans package, and pairwise comparisons on subscales with significant subgroup effects used Tukey’s adjustment. Effect sizes were calculated as Hedges’ g, using the average of the pre- and post-test standard deviations as the standardizer [24], to quantify the magnitude of pre–post change independently of statistical significance. Effect sizes were interpreted according to Cohen’s conventional thresholds, with values of 0.2, 0.5, and 0.8 representing small, medium, and large effects, respectively [25]. Statistical significance was set at *p* < 0.05.

## Results

**Fig. 1:**
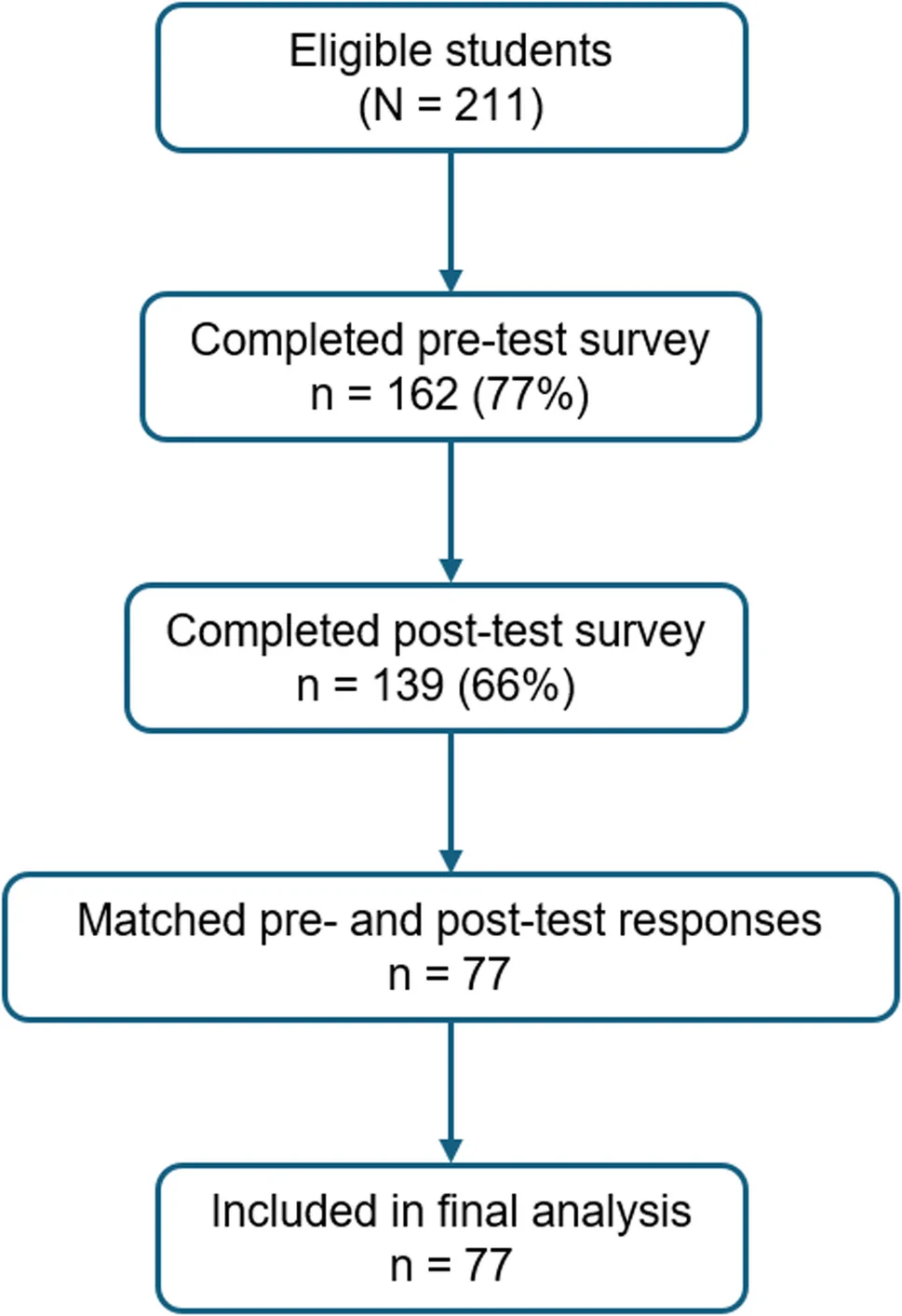
Flow diagram showing participant recruitment, survey completion, matching of anonymous questionnaires, and inclusion in the final analysis

Of the 211 students enrolled across the three academic levels, 162 completed the pre-test survey (response rate 77%) and 139 completed the post-test survey (response rate 66%). The use of anonymous self-generated identification codes enabled successful matching of pre- and post-test responses for 77 participants, who met the inclusion criteria and constituted the final analytic sample (Fig. 1). Table 1 summarizes their sociodemographic variables according to post-intervention responses. The majority of participants were female (*n* = 49; 64%), with 43% categorized as senior students (*n* = 33). Most respondents lived in urban areas (*n* = 67; 87%). Furthermore, 69% (*n* = 53) reported no prior involvement in community service.

**Table 1:** Sociodemographics of post-test respondents (*n* = 77)

Characteristics	Responders *n* (%)
Sex	
Male	28 (36)
Female	49 (64)
Academic level	
Junior (1st)	25 (32)
Intermediate (3rd)	19 (25)
Senior (4th and 5th)	33 (43)
Area of residence	
Urban	67 (87)
Rural	10 (13)
Prior experience in community work	
Yes	24 (31)
No	53 (69)

**Fig. 2:**
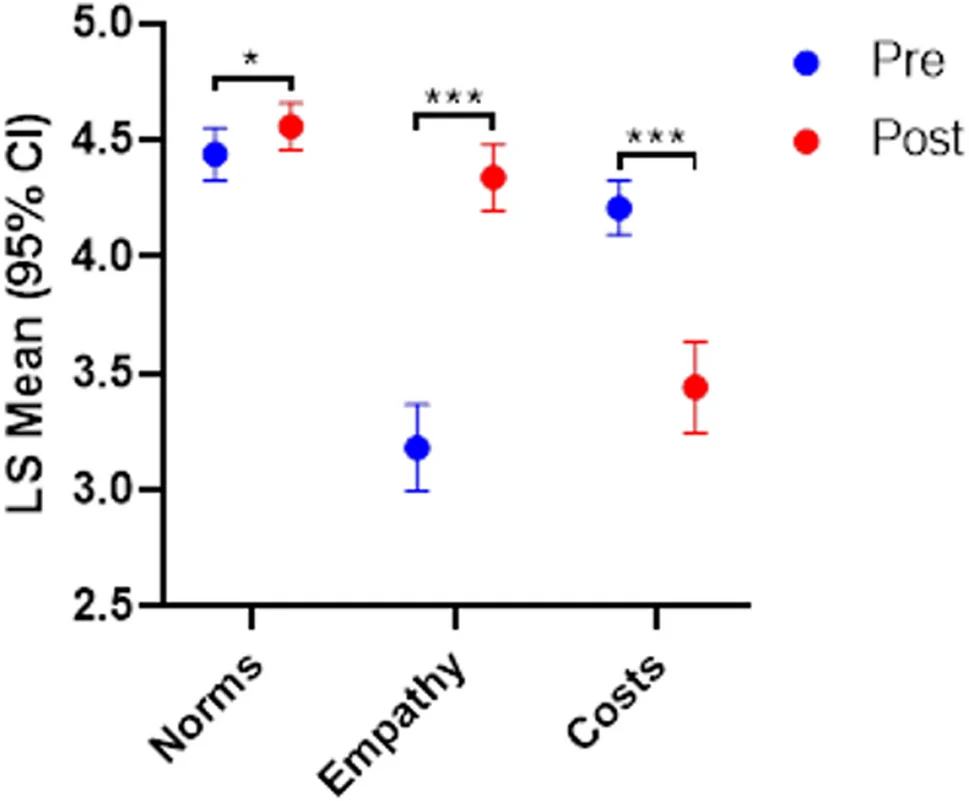
Pre- and post-intervention LS Mean with 95% confidence intervals for the three CSAS subscales showing statistically significant change: norms, empathy, and costs, in the matched sample (*n* = 77). Estimates were obtained from repeated-measures ANOVA. For the costs subscale, higher scores indicate greater perceived personal costs of community engagement, so the observed decrease represents a reduction in perceived barriers. *p < 0.05; ***p < 0.001

Of the ten CSAS subscales, three showed significant pre–post change — norms, empathy, and costs — whereas the remaining seven (awareness, actions, ability, connectedness, benefits, seriousness, and intention) did not (all *p* > 0.05) (Table 2). For these seven subscales, pre-test scores ranged from 4.13 to 4.54 and effect sizes were negligible (all |g| ≤ 0.17). Among the three subscales showing significant change (Fig. 2), norms increased from 4.44 (95% CI 4.33–4.55) to 4.56 (95% CI 4.45–4.66) at post-test (*p* = 0.035), corresponding to a small effect (Hedges’ g = 0.24; 95% CI 0.01–0.46). Empathy increased from 3.18 (95% CI 2.99–3.36) to 4.34 (95% CI 4.20–4.48; *p* < 0.001), corresponding to a large effect (g = 1.58; 95% CI 1.25–1.92).

**Table 2:** Changes between pre- and post test

Subscale	Occasion	LS Mean	95% CI	*P*-value	Hedges’ g (95% CI)
Awareness	Pre-test	4.54	4.41–4.66	0.811	-0.03 (-0.26, 0.19)
	Post-test	4.52	4.37–4.67		
Actions	Pre-test	4.45	4.33–4.57	0.901	0.01 (-0.21, 0.24)
	Post-test	4.45	4.33–4.58		
Ability	Pre-test	4.26	4.11–4.40	0.147	0.17 (-0.05, 0.40)
	Post-test	4.37	4.23–4.51		
Connectedness	Pre-test	4.22	4.08–4.35	0.667	-0.05 (-0.27, 0.18)
	Post-test	4.18	4.03–4.34		
Norms	Pre-test	4.44	4.33–4.55	**0.035**	0.24 (0.01, 0.46)
	Post-test	4.56	4.45–4.66		
Empathy	Pre-test	3.18	2.99–3.36	**< 0.001**	1.58 (1.25, 1.92)
	Post-test	4.34	4.20–4.48		
Costs	Pre-test	4.21	4.10–4.33	**< 0.001**	-1.12 (-1.41, -0.84)
	Post-test	3.44	3.24–3.63		
Benefits	Pre-test	4.50	4.39–4.60	0.681	-0.07 (-0.29, 0.15)
	Post-test	4.46	4.35–4.57		
Seriousness	Pre-test	4.17	4.03–4.32	0.631	-0.08 (-0.30, 0.15)
	Post-test	4.12	3.99–4.26		
Intention	Pre-test	4.13	3.94–4.31	0.839	0.03 (-0.19, 0.26)
	Post-test	4.15	3.99–4.31		

LS Mean, least-squares (estimated marginal) mean; CI, confidence interval. Estimates were obtained from repeated-measures ANOVA; LS Means, mean differences, and confidence intervals were computed using emmeans. The mean difference (post-test minus pre-test), expressed in the original 1–5 scale units, is the primary index of effect magnitude; Hedges’ g (standardizer: average of the pre- and post-test standard deviations) is reported to enable comparison with previous studies. Higher scores on the costs subscale indicate greater perceived personal costs of community engagement. Statistically significant changes (*p* < 0.05) are shown in bold

Scores on the costs subscale decreased after the intervention, with LS Mean scores dropping from 4.21 (95% CI 4.10–4.33) at pre-test to 3.44 (95% CI 3.24–3.63) at post-test (*p* < 0.001), corresponding to a large effect (g = − 1.12; 95% CI − 1.41 to − 0.84) and indicating a reduction in perceived barriers to community engagement.

Additional analyses examined whether the magnitude of change within the three significant subscales varied by academic level, sex, or prior experience with community work. For norms, no significant differences were found by academic level (*p* = 0.318), sex (*p* = 0.229), or prior experience (*p* = 0.813). For empathy, likewise, no significant differences were found by academic level (*p* = 0.322), sex (*p* = 0.070), or prior experience (*p* = 0.353).

**Fig. 3:**
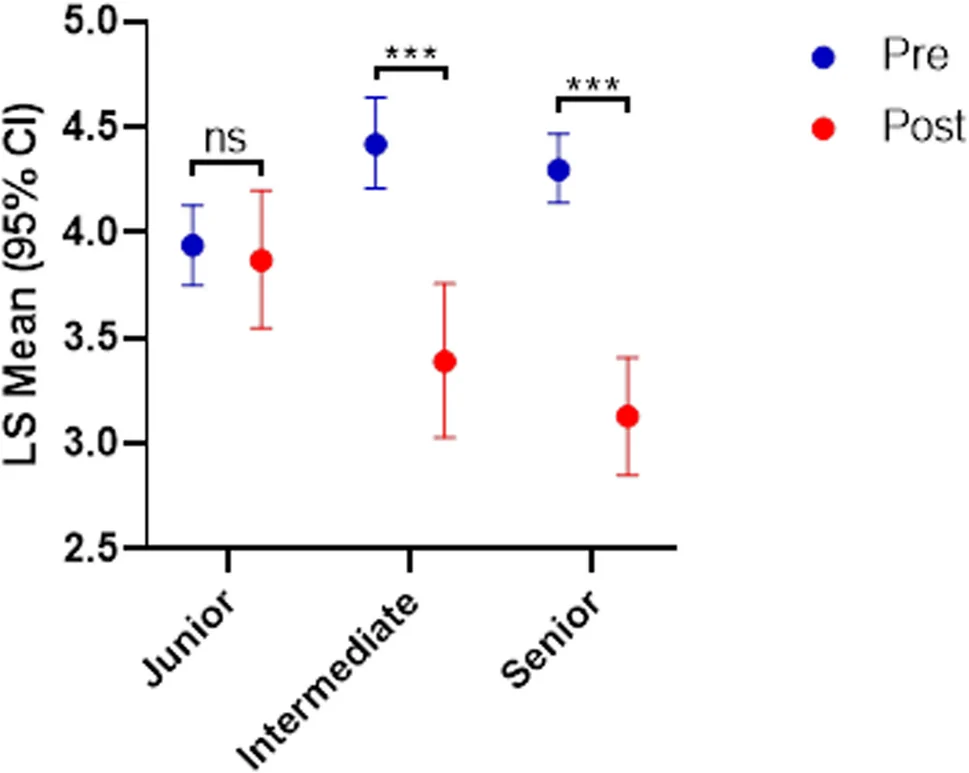
Pre- and post-intervention LS Mean with 95% confidence intervals for the costs subscale of the CSAS by academic level (Junior, *n* = 25; Intermediate, *n* = 19; Senior, *n* = 33). Estimates were obtained from repeated-measures ANOVA. Higher scores indicate greater perceived personal costs of community engagement, so a decrease represents a reduction in perceived barriers. This reduction was significantly smaller in junior than in intermediate and senior students (Tukey-adjusted). ***p < 0.001; ns, not significant

The magnitude of the reduction in perceived costs differed significantly by academic level (Fig. 3). Junior students showed no appreciable change (LS Mean 3.94 to 3.87; estimated change − 0.07; *p* = 0.716), whereas intermediate students (4.42 to 3.40; change − 1.03; *p* < 0.001) and senior students (4.30 to 3.13; change − 1.17; *p* < 0.001) both showed large reductions. Tukey-adjusted comparisons confirmed that the reduction was significantly smaller among junior students than among intermediate (*p* = 0.003) and senior students (*p* < 0.001), with no significant difference between the latter two groups (*p* = 0.846). No significant differences were found based on sex (*p* = 0.688) or prior experience with community service (*p* = 0.871).

## Discussion

Community engagement is increasingly recognized as an essential component of Dental Education, contributing to the development of ethical, social, and professional competencies [2, 7, 14]. In this study, participation in a Service-Learning experience was associated with significant improvements in Dental Students’ attitudes toward community service, particularly in the norms, empathy, and costs subscales. These findings are consistent with previous Service-Learning studies in dental education that used the same instrument [10, 13] and add data from a Latin American context, where such evidence remains scarce [14].

The observed increase in the norms subscale, although modest in magnitude (small effect), was associated with community-based activities and may reflect the internalization of prosocial principles and social responsibility. Service-Learning experiences appear to emphasize the value of assisting others, the importance of volunteering, and the need to contribute actively to community well-being. Previous research has similarly reported that structured community participation is associated with stronger normative prosocial conduct in health-profession students [7, 17].

One of the most notable results was the considerable increase observed in the empathy subscale, which showed a large effect. This competency is critical for patient-centered care and has been associated with better clinical outcomes, greater treatment adherence, and higher patient satisfaction [26–28]. These findings suggest that Service-Learning was associated with a deeper understanding of the social and emotional reality of the community. This is especially pertinent in the Chilean context, as students were exposed to neighborhoods with marked oral health disparities, which may have strengthened their empathy and reinforced their awareness of the socioeconomic determinants of health [14, 22].

In parallel, a particularly novel finding is the significant reduction in perceived costs, a subscale that reflects the personal sacrifices associated with participation, such as time taken away from studies, reduced leisure, and lost opportunities for paid work. This decrease suggests that direct community engagement was associated with a redefinition of participation as an integral component of professional development rather than a personal burden. The concomitant rise in empathy indicates a synergistic effect: as students gained a deeper understanding of the community’s reality, the subjective weight of personal costs diminished [29, 30]. Notably, this finding contradicts the findings of Bahammam and Bahammam [13], who used a similar instrument based on the eight-factor structure of the CSAS, in which empathy is not isolated as a separate subscale, and reported no significant change in the costs subscale. This contrast suggests that contextual or cultural factors specific to the Chilean setting, such as students’ exposure to marked oral health disparities, may have amplified this effect.

Despite these encouraging results, no significant changes were observed in seven of the ten subscales. This may be attributable to a ceiling effect, as baseline scores on these subscales ranged from 4.13 to 4.54 out of 5, indicating that students held highly favorable attitudes toward community service before the intervention [31]. In psychometric terms, when baseline scores approach the upper limit of a scale, the instrument’s capacity to detect further change is inherently limited, regardless of the intervention’s actual impact. In addition, the heterogeneity of the intervention in terms of duration, intensity, and activity type may have limited its overall effect. Previous research indicates that structured, longitudinal community-based interventions tend to yield more consistent benefits across learning domains [32, 33].

The magnitude of change did not differ significantly by sex in any subscale, suggesting that the attitudinal benefits associated with the experience were comparable for female and male students. This is consistent with [10] Coe et al., who, using the same instrument, found that student characteristics such as sex did not significantly affect attitudes toward community service. Likewise, the magnitude of change did not differ by prior experience in community work (all interaction *p* > 0.05), suggesting that the observed changes were not driven solely by students already inclined toward community engagement. Nonetheless, self-selection at the level of study enrollment cannot be excluded, as students who chose to participate may differ systematically from those who did not.

Subgroup analysis showed that the reduction in perceived costs was concentrated among students at more advanced stages of training, whereas junior students showed no appreciable change. This pattern may reflect that advanced students tend to be more confident in their clinical and communication skills, which could allow a community experience to translate more readily into a lower perception of personal barriers [30, 34]. Junior students, by contrast, often experience greater uncertainty when first exposed to clinical and community settings [35], which may offset any reduction in perceived costs. Because the reduction was minimal among junior students, a brief, isolated exposure may be insufficient to reduce perceived barriers at early stages and may instead make the personal demands of community work more salient. The curricular implication is that early community-based experiences should not be assumed to be self-sufficient, but rather scaffolded through longitudinal, progressively structured activities with adequate supervision and reflection.

By contrast, the gains in empathy and norms did not vary by academic level, indicating that these benefits were not restricted to advanced students and supporting the early inclusion of community-based activities in the Dental Curriculum. Taken together, these results suggest that although early exposure is valuable for cultivating empathy and prosocial norms, reducing the perceived personal barriers to community engagement at junior levels may require more sustained curricular support.

From an educational perspective, these findings support the inclusion of Service-Learning into the Dental Curriculum as a strategy for promoting holistic training. The World Health Organization has emphasized the need for socially oriented health education in addressing health inequities [36]. In this context, Service-Learning enables the integration of technical knowledge with a broader understanding of the socioeconomic determinants of health [37], an approach still underdeveloped in Dental Education in Chile. Beyond individual learning outcomes, Community-Based Dentistry Education has been associated with broader public health benefits, such as improved access to dental care services and greater health literacy among underserved communities [38]. These benefits are particularly relevant in Chile, where persistent socioeconomic disparities in access to oral health care underscore the need for socially responsible dental professionals [22]. Plessas et al., [14] reported that community-engaged education fosters empathy, an understanding of the social determinants of health, and a commitment to equity; however, none of the studies included in their review were conducted in Latin America, revealing an important geographical gap. The present findings contribute data from Chile and support the global relevance of Service-Learning as an approach for training socially responsible dental practitioners in settings marked by substantial health disparities.

Several limitations should be considered when interpreting these results. The pre–post design without a control group restricts causal inference: because attitudes were not compared against an untreated group, the observed changes cannot be attributed solely to the intervention, and external factors —such as academic maturation, concurrent clinical experiences, or curricular progression— may have contributed. Consequently, although the results indicate a positive association between participation in Service-Learning and improvements in empathy, norms, and perceived costs, a direct causal relationship cannot be established. The convenience sampling strategy may have introduced selection bias, as students with greater initial interest in Service-Learning may have been more likely to participate and complete both measurements, potentially underrepresenting less motivated students and limiting the generalizability of the findings. Third, although 162 students completed the pre-test and 139 the post-test, only 77 responses could be matched and included in the final analysis, owing to unmatched anonymous codes and the exclusion of incomplete instruments. Because participation was voluntary and non-mandatory, this attrition is expectable but methodologically relevant: it may have reduced the precision of the estimates and increased the risk of bias if the students excluded differed systematically from those retained. These findings should therefore be interpreted with caution and not broadly generalized without considering these constraints.

Future research should consider controlled or randomized designs where feasible, together with longitudinal follow-up to assess the persistence of attitudinal change over time. Multicenter and probabilistic samples would help increase the generalizability of the findings across dental programs and sociocultural contexts. In addition, mixed-methods studies integrating quantitative and qualitative approaches could provide a deeper understanding of the mechanisms through which community experiences influence empathy, social responsibility, perceived costs, and the construction of professional identity in dental students.

## Conclusions

The present findings indicate that participation in Service-Learning activities was associated with positive changes in relevant attitudinal dimensions among dental students, specifically empathy, social norms, and perceived costs. These results underscore the value of integrating community-based experiences into Dental Schools to support the development of more ethical, reflective, and patient-centered practitioners. In Chile, where socioeconomic disparities in oral health access persist, these findings highlight the importance of formally incorporating Service-Learning into the Dental Curriculum as a means of training professionals who possess not only clinical competencies but also the social awareness and ethical commitment required to address the oral health needs of underserved populations.

## Supplementary Information

Additional file 1.

Additional file 2.

Additional file 3.

## Data Availability

The data that support the findings of this study are available on request from the corresponding author. The data are not publicly available due to privacy or ethical restrictions.
