# Supplementary material for: Dental students’ attitudes toward community service: a pre–post survey study of a service-learning experience in Chile
Source: BMC Med Educ. 2026 Aug 12;26:1561. doi: 10.1186/s12909-026-10109-w (PMC13628780; doi:10.1186/s12909-026-10109-w)
Supplement: Supplementary file 1 [file 12909_2026_10109_MOESM1_ESM.docx]

**Community Service Attitudes Scale (CSAS): original English items and the Spanish version administered in this study**

The instrument used in this study is the Community Service Attitudes Scale (CSAS), a previously published, Likert-type instrument developed by Shiarella, McCarthy and Tucker (2000) to measure college students' attitudes toward community service. The scale is organized into ten components grounded in Schwartz's helping-behaviour model: Awareness, Actions, Ability, Connectedness, Norms, Empathy, Costs, Benefits, Seriousness, and Intention.

For the present study, the original English items were translated into Spanish and reviewed by a bilingual native speaker to ensure semantic equivalence. The table below presents each original English item alongside the Spanish version as administered, grouped by component. Responses were recorded on a five-point Likert scale (1 = strongly disagree to 5 = strongly agree), in contrast to the seven-point scale used in the original instrument.

| **Original item (English), Shiarella et al. (2000)** | **Spanish version as administered** |
| --- | --- |
| **Awareness** | **Conciencia** |
| Community groups need our help. | Los grupos comunitarios necesitan nuestra ayuda. |
| There are people in the community who need help. | Hay personas en la comunidad que necesitan ayuda. |
| There are needs in the community. | Hay necesidades en la comunidad. |
| There are people who have needs which are not being met. | Hay personas cuyas necesidades no están siendo satisfechas. |
| **Actions** | **Acciones** |
| Volunteer work at community agencies helps solve social problems. | El trabajo voluntario en organizaciones comunitarias ayuda a resolver problemas sociales. |
| Volunteers in community agencies make a difference, if only a small difference. | Los voluntarios en organizaciones comunitarias marcan la diferencia, aunque sea pequeña. |
| College student volunteers can help improve the local community. | Los estudiantes universitarios que hacen voluntariado pueden ayudar a mejorar la comunidad local. |
| Volunteering in community projects can greatly enhance the community's resources. | Participar como voluntario en proyectos comunitarios puede mejorar significativamente los recursos de la comunidad. |
| The more people who help, the better things will get. | Cuantas más personas colaboren, mejorarán las cosas. |
| **Ability** | **Capacidad** |
| Contributing my skills will make the community a better place. | Aportar mis habilidades hará de la comunidad un lugar mejor. |
| My contribution to the community will make a real difference. | Mi contribución a la comunidad marcará una verdadera diferencia. |
| I can make a difference in the community. | Puedo marcar la diferencia en la comunidad. |
| **Connectedness** | **Conexión** |
| I am responsible for doing something about improving the community. | Soy responsable de hacer algo para mejorar la comunidad. |
| It is my responsibility to take some real measures to help others in need. | Es mi responsabilidad tomar medidas concretas para ayudar a quienes lo necesitan. |
| It is important to me to have a sense of contribution and helpfulness through participating in community service. | Es importante para mí sentir que contribuyo y soy útil a través del servicio comunitario. |
| It is important to me to gain an increased sense of responsibility from participating in community service. | Es importante para mí desarrollar un mayor sentido de responsabilidad mediante la participación en el servicio comunitario. |
| I feel an obligation to contribute to the community. | Siento la obligación de contribuir a la comunidad. |
| Other people deserve my help. | Otras personas merecen mi ayuda. |
| **Norms** | **Normas** |
| It is important to help people in general. | Es importante ayudar a las personas en general. |
| Improving communities is important to maintaining a quality society. | Mejorar las comunidades es fundamental para mantener una sociedad de calidad. |
| Our community needs good volunteers. | Nuestra comunidad necesita buenos voluntarios. |
| All communities need good volunteers. | Todas las comunidades necesitan buenos voluntarios. |
| It is important to provide a useful service to the community through community service. | Es importante brindar un servicio útil a la comunidad a través del servicio comunitario. |
| **Empathy** | **Empatía** |
| When I meet people who are having a difficult time, I wonder how I would feel if I were in their shoes. | Cuando conozco a personas que están pasando por un momento difícil, me pregunto cómo me sentiría si estuviera en su lugar. |
| I feel bad that some community members are suffering from a lack of resources. | Me siento mal al saber que algunos miembros de la comunidad sufren por la falta de recursos. |
| I feel bad about the disparity among community members. | Me entristece la desigualdad entre los miembros de la comunidad. |
| **Costs** | **Costos percibidos** |
| I would have less time for my schoolwork. | Tendría menos tiempo para mis estudios. |
| I would have forgone the opportunity to make money in a paid position. | Habría renunciado a la oportunidad de ganar dinero en un trabajo remunerado. |
| I would have less energy. | Tendría menos energía. |
| I would have less time to work. | Tendría menos tiempo para trabajar. |
| I would have less free time. | Tendría menos tiempo libre. |
| I would have less time to spend with my family. | Tendría menos tiempo para pasar con mi familia. |
| **Benefits** | **Beneficios percibidos** |
| I would be contributing to the betterment of the community. | Estaría contribuyendo a la mejora de la comunidad. |
| I would experience personal satisfaction knowing that I am helping others. | Experimentaría satisfacción personal al saber que estoy ayudando a los demás. |
| I would be meeting other people who enjoy community service. | Conocería a otras personas que disfrutan del servicio comunitario. |
| I would be developing new skills. | Desarrollaría nuevas habilidades. |
| I would make valuable contacts for my professional career. | Establecería contactos valiosos para mi carrera profesional. |
| I would gain valuable experience for my resume. | Adquiriría experiencia valiosa para mi currículum. |
| **Seriousness** | **Seriedad** |
| Lack of participation in community service will cause severe damage to our society. | La falta de participación en el servicio comunitario causará un daño grave a nuestra sociedad. |
| Without community service, today's disadvantaged citizens have no hope. | Sin el servicio comunitario, los ciudadanos desfavorecidos de hoy no tienen esperanza. |
| Community service is necessary to making our communities better. | El servicio comunitario es necesario para mejorar nuestras comunidades. |
| It is critical that citizens become involved in helping their communities. | Es fundamental que los ciudadanos se involucren en ayudar a sus comunidades. |
| Community service is a crucial component of the solution to community problems. | El servicio comunitario es un componente crucial de la solución a los problemas comunitarios. |
| **Intention to engage in community service** | **Intención de participación en servicio comunitario** |
| I want to do this (service-learning) activity. | Quiero realizar esta actividad (aprendizaje-servicio). |
| I will participate in a community service project in the next year. | Participaré en un proyecto de servicio comunitario durante el próximo año. |
| Would you seek out an opportunity to do community service in the next year? | ¿Buscarías una oportunidad para hacer servicio comunitario en el próximo año? |

Notes. The instrument administered comprised 46 items across the ten CSAS components. Sociodemographic items (age, sex, area of residence, prior experience in community work, and academic year) were collected separately and are not reproduced here.

**Reference**

Shiarella, A. H., McCarthy, A. M., & Tucker, M. L. (2000). Development and construct validity of scores on the Community Service Attitudes Scale. Educational and Psychological Measurement, 60(2), 286–300. <https://doi.org/10.1177/00131640021970510>
