# Supplementary material for: Dental students’ attitudes toward community service: a pre–post survey study of a service-learning experience in Chile
Source: BMC Med Educ. 2026 Aug 12;26:1561. doi: 10.1186/s12909-026-10109-w (PMC13628780; doi:10.1186/s12909-026-10109-w)
Supplement: Supplementary file 2 [file 12909_2026_10109_MOESM2_ESM.docx]

**Additional file 2.** Internal consistency (Cronbach’s α) of the CSAS subscales at the pilot.

| **CSAS subscale** | **Items (n)** | **Cronbach’s α** |
| --- | --- | --- |
| Awareness | 4 | 0.548 |
| Actions | 5 | 0.853 |
| Ability | 3 | 0.924 |
| Connectedness | 6 | 0.768 |
| Norms | 5 | 0.886 |
| Empathy | 3 | 0.774 |
| Costs | 6 | 0.835 |
| Benefits | 6 | 0.965 |
| Seriousness | 5 | 0.825 |
| Intention | 3 | 0.779 |
| **Overall scale** | **46** | **0.929** |
