# Supplementary material for: Dental students’ attitudes toward community service: a pre–post survey study of a service-learning experience in Chile
Source: BMC Med Educ. 2026 Aug 12;26:1561. doi: 10.1186/s12909-026-10109-w (PMC13628780; doi:10.1186/s12909-026-10109-w)
Supplement: Supplementary file 3 [file 12909_2026_10109_MOESM3_ESM.docx]

**Additional file 3.** Internal consistency (Cronbach’s α) of the CSAS subscales at pre-test and post-test.

| **CSAS subscale** | **Items (n)** | **Pre-test α** | **Post-test α** |
| --- | --- | --- | --- |
| Awareness | 4 | 0.827 | 0.920 |
| Actions | 5 | 0.853 | 0.919 |
| Ability | 3 | 0.852 | 0.878 |
| Connectedness | 6 | 0.839 | 0.887 |
| Norms | 5 | 0.796 | 0.928 |
| Empathy | 3 | 0.709 | 0.825 |
| Costs | 6 | 0.758 | 0.916 |
| Benefits | 6 | 0.804 | 0.910 |
| Seriousness | 5 | 0.849 | 0.852 |
| Intention | 3 | 0.908 | 0.877 |
| **Overall scale** | **46** | **0.885** | **0.957** |
